# Independent somatic *TP53* mutations in blood and tumor mimicking Li-Fraumeni syndrome in a 94-year-old man

**DOI:** 10.1038/s41698-026-01544-5

**Published:** 2026-06-08

**Authors:** Chuan Gao, Vikas Rai, Donna Wong, Zarina Yelskaya, Silvana Ostafi, Joshua Somar, Neal Cody, Panieh Terraf, Maksym Misyura, Ozge Ceyhan-Birsoy, Diana Mandelker

**Affiliations:** Department of Pathology and Laboratory Medicine, Memorial Sloan Kettering Cancer Center, New York, NY USA

**Keywords:** Cancer, Genetics, Oncology

## Abstract

Accurately determining the origin of *TP53* variants detected in blood is essential as such variants may arise from the constitutional state or clonal hematopoiesis (CH). While paired tumor-blood sequencing is widely used to distinguish somatic from constitutional events, convergent acquisition of identical *TP53* hotspot mutations in tumor and blood can obscure variant origin and complicate interpretation. Here we report a 94-year-old patient with the *TP53* c.524 G > A (p.Arg175His) pathogenic variant identified in both peripheral blood and tumor tissue, initially raising concern for Li-Fraumeni syndrome (LFS). Given the patient’s atypical LFS clinical presentations, multi-tissue confirmatory analysis was performed. Normal bladder tissue and skin biopsy each showed trace mutant signal, and fingernail DNA was negative. These findings support independent somatic acquisition of the same *TP53* hotspot mutation in hematopoietic and tumor lineages. This case highlights the importance of multi-tissue confirmatory testing to accurately determine the origin of *TP53* variants in cancer patients.

## Introduction

Li-Fraumeni syndrome (LFS) is a rare autosomal dominant cancer-predisposition disorder caused by constitutional pathogenic variants in *TP53*. Affected individuals have elevated lifetime risks for multiple malignancies including soft-tissue and osteosarcomas, breast cancer, adrenocortical carcinoma, brain tumors, and leukemia^1,2^. For classic LFS, the lifetime cancer risk exceeds 80% by age 70^3^. In an international cohort of 4028 individuals with LFS, the cumulative risk of any cancer by age 50 years was 92.4% in women and 59.7% in men^4^.

In contrast to constitutional *TP53* mutations, somatic variants confined to hematopoietic cells, also known as clonal hematopoiesis (CH) or clonal hematopoiesis of indeterminate potential (CHIP), are increasingly recognized in aging populations^5^. *TP53* is among the most frequent CH genes, with CH-associated *TP53* variants detected in approximately 3% of older adults over 80 years in the general population and up to 10% of cancer patients^6–9^. Although CH confers a modestly increased risk for hematologic malignancy and cardiovascular disease, its clinical implications differ fundamentally from those of constitutional *TP53* pathogenic variants that cause LFS^6,10,11^.

Most CH-associated variants are detected at relatively low VAFs, which can complicate distinction between CH and constitutional mosaicism^12^. Paired tumor-normal sequencing can help clarify variant origin: constitutional mosaic variants are often enriched in tumor and display higher VAFs, whereas CH-derived variants are confined to hematopoietic cells and are typically absent from tumor tissue or detected only at low levels due to tumor-infiltrating blood cells. However, CH-derived variants can sometimes expand to VAFs within the expected heterozygous range, thereby confounding interpretation in clinical germline genetic testing^13,14^. For example, in a study of 46 individuals with pathogenic *TP53* variants detected in blood at VAFs of 30-70%, 32.6% (15/46) were absent in paired fibroblast samples, supporting a non-constitutional origin in a substantial subset^15^. Distinguishing CH from constitutional variants is therefore essential for accurate variant interpretation.

Paired tumor-blood sequencing, such as in the MSK-IMPACT program, is widely used to differentiate somatic from constitutional variants in cancer patients receiving genetic testing^16^. Because CH mutations are confined to hematopoietic cells, they are typically absent in tumor tissue, allowing comparison of variant allele fractions (VAFs) across tissues to inform origin^17^. However, certain *TP53* hotspot mutations, such as p.Arg175His, are recurrent as both CH and tumor somatic mutations, raising the possibility of independent acquisition of the same variant in both blood and tumor. Such coincidences, while rare, can generate false suspicion for hereditary cancer syndromes and prompt unnecessary anxiety, surveillance, or familial testing.

Here, we describe a 94-year-old man with bladder cancer, in whom the same *TP53* c.524 G > A (p.Arg175His) pathogenic variant was identified in both blood and tumor at high allele fraction. This variant has been reported to segregate with LFS-associated cancers with high penetrance, lead to non-functional transactivation and loss of growth suppression activity in functional assays, and is thus classified as pathogenic for LFS by ClinGen *TP53* Variant Curation Expert Panel^18^. Its detection in both blood and tumor therefore initially raised suspicion for LFS. Multi-tissue analysis, including DNA from a skin sample, adjacent normal tissue and fingernails, demonstrated that the variant represented clonal hematopoiesis in blood and an independent somatic driver mutation in the tumor, rather than a constitutional event. This case highlights the diagnostic complexity of interpreting *TP53* variants across tissues and underscores the importance of multi-tissue validation and clinical correlation in precision oncology.

## Results

### Case report

A 94-year-old man of European ancestry was diagnosed with stage IIIB muscle-invasive urothelial carcinoma of the bladder at age 92. He underwent transurethral resection of bladder tumor (TURBT) followed by systemic therapy with enfortumab vedotin plus pembrolizumab, and subsequent chemoradiation. At the time of last follow-up, approximately two years after diagnosis, the patient remained clinically stable with no evidence of recurrent or new malignancy.

**Fig. 1:**
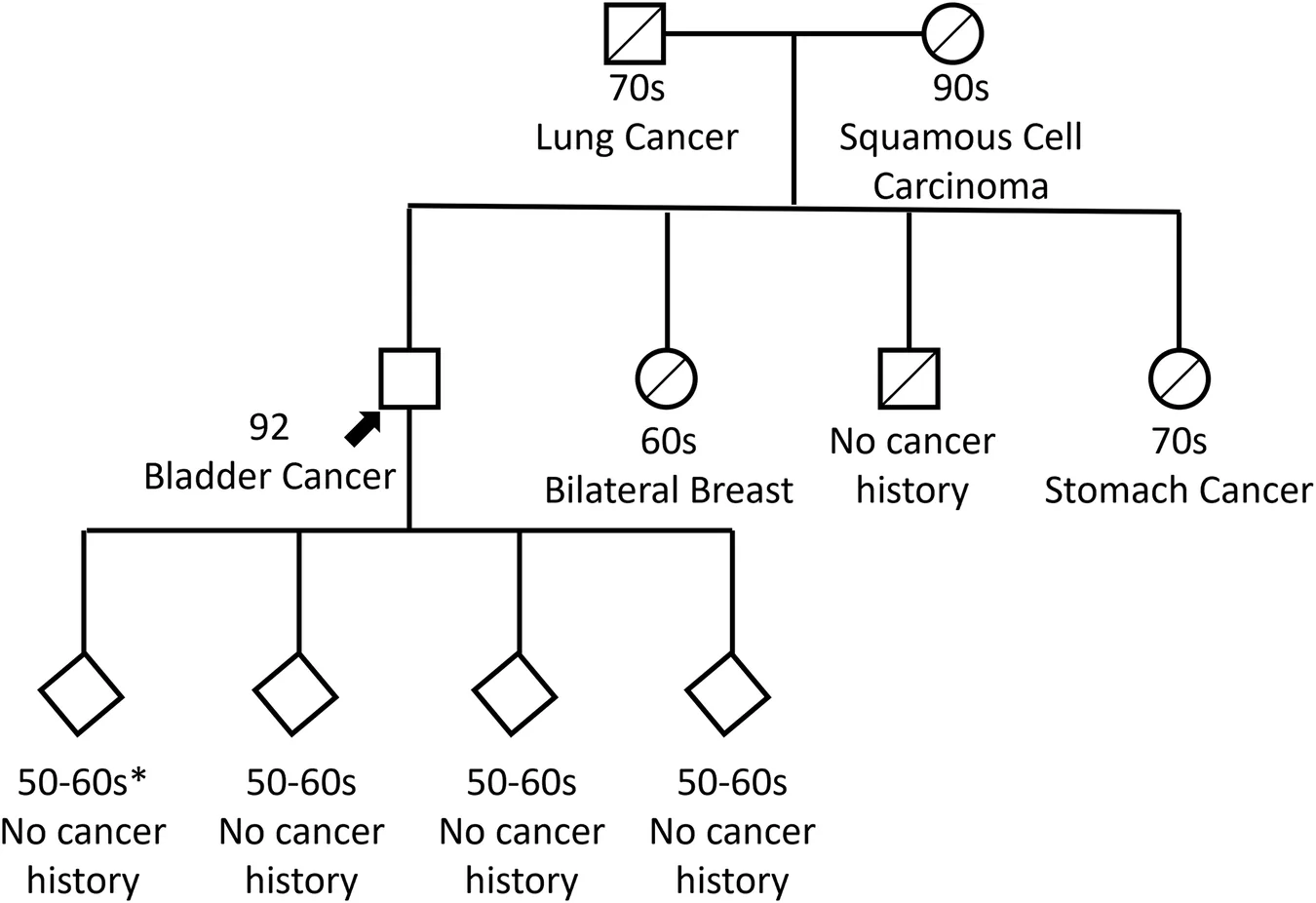
Pedigree of the proband and family history of cancer. The proband (arrow) was diagnosed with bladder urothelial carcinoma at age 92. Affected relatives include the father with lung cancer in his 70 s, mother with squamous cell carcinoma in her 90 s, one sister with bilateral breast cancer in her 60 s, and another sister with stomach cancer in her 70 s. The asterisk indicates the offspring who tested negative for the *TP53* c.524 G > A (p.Arg175His) variant in blood-derived DNA.

The patient’s medical history was notable only for benign prostatic hyperplasia, and there was no prior malignancy. Family history revealed that his father developed lung cancer in his 70 s; his mother was diagnosed with squamous cell carcinoma in her 90 s; one sister had bilateral breast cancer in her 60 s; and another sister had stomach cancer in her 70 s. No family member had early-onset or Li-Fraumeni spectrum cancers (Fig. 1).

**Fig. 2:**
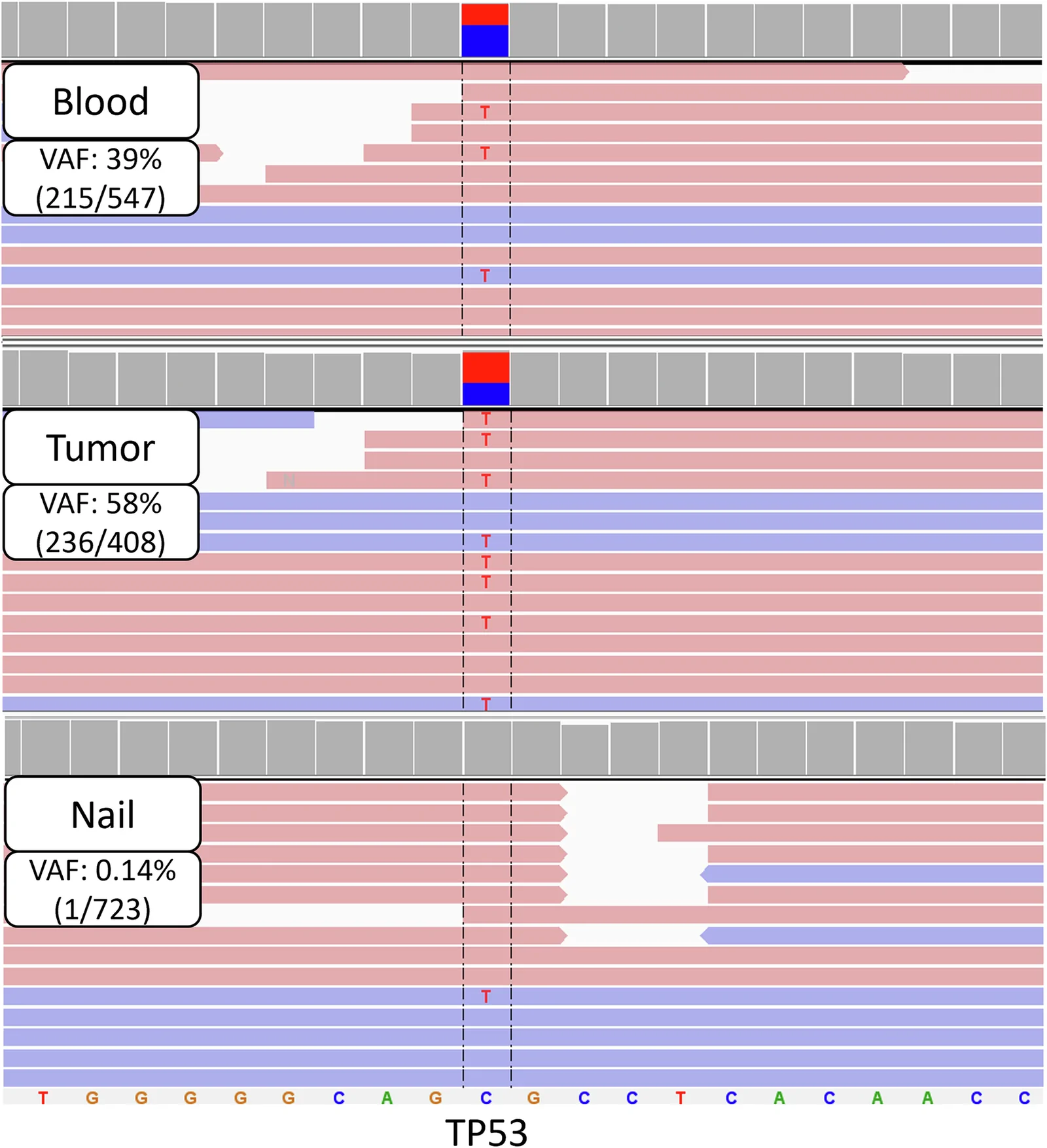
Integrative genomics viewer (IGV) visualization of next-generation sequencing (NGS) results for *TP53* c.524 G > A (p.Arg175His) in blood, tumor, and fingernail DNA. NGS reads demonstrate the *TP53* c.524 G > A (p.Arg175His) variant in both blood (215/547; VAF: 39%) and tumor (236/408; VAF: 58%) samples. In contrast, only a single variant read was detected among 723 total reads in fingernail DNA (1/723; VAF: 0.14%), a finding likely consistent with background sequencing error rather than true variant presence.

Comprehensive next-generation sequencing (NGS) of the peripheral blood using MSK-IMPACT identified a *TP53* pathogenic variant, c.524 G > A (p.Arg175His), at a VAF of 39% (215/547) (Fig. 2**;** Table 1). Matched tumor testing from the formalin-fixed, paraffin-embedded (FFPE) specimen (tumor purity: 43%) revealed the same *TP53* variant at a VAF of 58% (236/408) (Fig. 2; Table 1). Notably, comparison of the overall molecular profiles in blood and tumor did not support circulating tumor DNA (ctDNA) contamination of the blood specimen, as no additional tumor-associated variants were detected in blood. Therefore, the concurrent detection of this variant in both blood and tumor initially raised concern for LFS.

**Table 1:** Summary of *TP53* c.524 G > A (p.Arg175His) variant allele fractions across tested tissues

Tissue	Assay	Variant Allele Fraction	Interpretation
Blood	NGS	39% (215/547)	Clonal hematopoiesis
Bladder tumor(FFPE)	NGS	58% (236/408)	Tumor somatic mutation
Skin biopsy (FFPE)	Sanger	<5% (by visual estimation)	Likely presence of small amount of hematologic cells.
Adjacent normal tissue (FFPE)	Sanger	<5% (by visual estimation)	Likely presence of small amount of hematologic or tumor cells.
Fingernail DNA	NGS	0.14% (1/723)	Negative^a^ germline DNA

^a^A single mutant read detected in nail DNA was most consistent with sequencing background noise rather than true mosaicism and was therefore interpreted as negative.

### Subsequent multi-tissue confirmatory testing

**Fig. 3:**
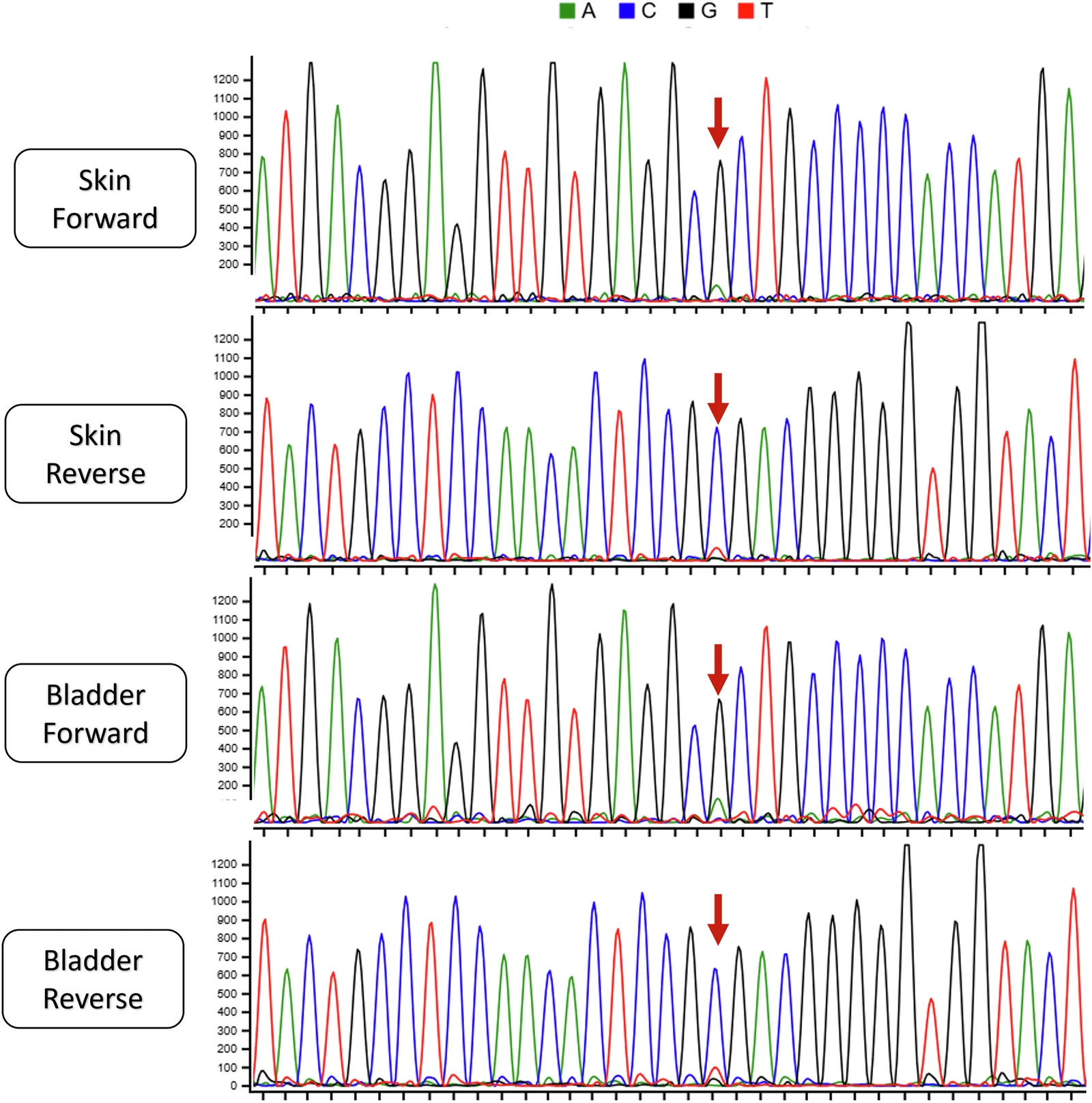
Sanger sequencing of *TP53* c.524 G > A (p.Arg175His) in skin biopsy and normal bladder tissue adjacent to the tumor. Electropherograms show minor mutant peaks in both skin and normal bladder tissue, consistent with a very low variant fraction ( < 5% by visual estimation), likely reflecting hematopoietic or tumor DNA contamination.

Given the patient’s advanced age, absence of LFS-associated malignancies, and non-suggestive family history, a diagnosis of LFS would be highly unexpected for a *TP53* variant typically associated with early-onset, high-penetrance cancer predisposition. Accordingly, confirmatory multi-tissue testing was performed to clarify the variant’s origin. DNA extracted from both normal bladder tissue adjacent to the tumor and from a skin biopsy of the patient’s left back revealed minor mutant peaks on Sanger sequencing, each consistent with a very low variant fraction ( < 5% by visual estimation; Fig. 3). These low-level signals likely reflect hematopoietic cell DNA contamination rather than true mosaicism as some hematopoietic cells are present in micro-dissected tissue specimens. Fingernail DNA has been demonstrated to serve as a reliable source of germline DNA that lacks hematopoietic-derived somatic variants in individuals with clonal hematopoiesis or hematologic neoplasms^19,20^. Accordingly, fingernail DNA was analyzed by NGS and revealed a single variant read among 723 total reads (1/723, VAF 0.14%; Fig. 2). Given the high GC content surrounding the *TP53* c.524 G > A locus and the expected background noise of NGS, this isolated read is interpreted as a sequencing artifact rather than evidence of low-level mosaicism. One of the patient’s offspring also tested negative for *TP53* c.524 G > A in blood-derived DNA.

Together, these results indicated that the *TP53* c.524 G > A (p.Arg175His) variant detected in blood most likely represented clonal hematopoiesis, while the same variant identified in the tumor arose independently as a somatic mutation. Thus, the variant was inconsistent with a constitutional *TP53* variant associated with LFS.

## Discussion

To our knowledge, this is the first reported case demonstrating independent acquisition of the same *TP53* mutation as both clonal hematopoiesis in blood and a somatic mutation in the tumor. The *TP53* c.524 G > A (p.Arg175His) variant is a well-established cancer hotspot^21^. As of October 2025, this alteration has been identified as a clonal hematopoiesis-associated variant in 0.11% (94/82,668) of individuals and as a somatic mutation in 1.8% (63/3,443) of bladder cancer cases analyzed by MSK-IMPACT. Located within the DNA-binding domain, p.Arg175His is among the most frequently reported *TP53* pathogenic variants and exerts dominant-negative and loss-of-function effects^22–24^.

Although its presence in both blood and tumor initially suggested LFS, several observations raised doubts about that interpretation: (1) the patient’s advanced age and absence of early-onset or LFS-spectrum cancers; (2) a family history inconsistent with LFS; and (3) absence of the variant in fingernail DNA. These findings support an interpretation of clonal hematopoiesis in blood and an independent somatic mutation in the tumor, representing a rare example of independent acquisition of the same variant.

Recent studies suggest tumor-infiltrating clonal hematopoiesis (TI-CH) is common and may result in low-level detection of CH-associated variants within tumor sequencing data^25^. However, several features in this case strongly support that the tumor *TP53* variant was not a result of TI-CH. Notably, the tumor VAF (58%) exceeded that of the peripheral blood (39%), contradicting the expected dilution effect of infiltrating hematopoietic cells. Furthermore, an independent CH variant (TET2 c.4063 G > C p.Ala1355Pro) was detected at 35% VAF in blood but only 5% in the tumor, suggesting only minimal infiltration. Together, these findings support independent somatic acquisition of the same *TP53* hotspot mutation in hematopoietic and tumor lineages rather than high-burden TI-CH.

This case exemplifies a growing interpretive challenge in precision oncology. The increased sensitivity of modern sequencing assays has led to more frequent detections of CH variants, particularly in older individuals. Recent studies suggest that CH is present in more than 60% of individuals over age 80, most frequently involving *DNMT3A* and *TET2*^8^. *TP53* is also among the most frequent CH genes, observed in approximately 3% of individuals over 80 years of age and up to 10% of patients with cancer^8,9^. *TP53*-associated CH has been linked to diverse outcomes beyond myeloid neoplasm mortality and, when combined with environmental exposures, is associated with markedly increased disease-specific mortality^6^. Paired tumor-blood sequencing platforms such as MSK-IMPACT are invaluable for distinguishing CH, constitutional, and tumor-specific variants. However, when an identical alteration is detected in both blood and tumor samples in a patient whose clinical features are inconsistent with a constitutional syndrome, additional multi-tissue testing is essential to determine the variant’s origin.

Misinterpreting a CH-related *TP53* variant as LFS can have major clinical and psychosocial consequences, including unnecessary counseling, lifelong cancer surveillance, reproductive anxiety, and cascade testing among relatives. Conversely, failing to recognize a true constitutional variant could result in missed opportunities for early detection and risk management.

One limitation of the study is the absence of additional tissue specimens for further confirmation. As a result, the possibility of a mosaic *TP53* variant with strong tissue specificity cannot be definitively ruled out. However, given the patient’s advanced age and inconsistent LFS features, this possibility is considered exceedingly unlikely.

In summary, independent *TP53* mutations arising in blood and tumor can mimic constitutional pathogenic variants and generate false suspicion for Li-Fraumeni syndrome. Although rare, this scenario highlights an important diagnostic limitation of tumor-normal (blood) paired sequencing, which is increasingly used in routine precision oncology practice. Identical hotspot mutations detected in both compartments do not necessarily indicate a constitutional event, particularly in older individuals with age-related clonal hematopoiesis. Comprehensive multi-tissue evaluation, integrated with careful clinical correlation and family history assessment, may be needed to avoid misclassification and its significant clinical and psychosocial consequences. This case emphasizes the need for cautious interpretation of shared tumor-blood variants to ensure accurate diagnosis and appropriate patient management.

## Methods

### Patient enrollment and ethical compliance

The patient was enrolled in an IRB-approved research protocol at Memorial Sloan Kettering Cancer Center (MSKCC IRB protocol #12-245; ClinicalTrials.gov identifier NCT01775072). The study was conducted in accordance with the ethical principles of the Declaration of Helsinki and applicable regulatory requirements. The patient provided written informed consent for genetic sequencing, research participation, and the publication of de-identified data.

### Next-generation sequencing and data analysis

Genetic analysis of blood, tumor, and fingernail DNA was performed using the MSK-IMPACT assay, a hybridization capture-based targeted next-generation sequencing (NGS) platform authorized by the U.S. Food and Drug Administration (FDA) and approved by the New York State Department of Health for clinical use. Detailed workflow, performance characteristics, and computational methods have been previously described, including the original MSK-IMPACT validation, the germline analysis framework, and its application to hematologic neoplasms^16,26,27^. Tumor purity was calculated using FACET^28^.

### Orthogonal validation and multi-tissue analysis

For confirmatory testing, benign bladder tissue adjacent to the tumor was identified by a pathologist, micro-dissected from FFPE slides, and processed for DNA extraction. A skin biopsy from the left back was collected, and DNA was isolated from FFPE slides using standard protocols. Variant detection in adjacent normal bladder tissue and skin biopsy specimens was assessed by Sanger sequencing.

### Variant interpretation

Variant interpretation followed ACMG classification guidelines^29^.

## Data Availability

The datasets generated and/or analyzed during the current study are not publicly available due to ethical and privacy restrictions related to human genomic data. De-identified data may be made available from the corresponding author on reasonable request, subject to institutional review and approval.
